# *N*-(4-Chloro­butano­yl)-*N*′-(2,5-dimeth­oxy­phen­yl)thio­urea

**DOI:** 10.1107/S1600536811036002

**Published:** 2011-09-14

**Authors:** M. Sukeri M. Yusof, Norafiqah R. Azmi, Bohari M. Yamin

**Affiliations:** a Department of Chemical Sciences, Faculty of Science and Technology, Universiti Malaysia Terengganu, 21030 Kuala Terengganu, Terengganu, Malaysia; b School of Chemical Sciences and Food Technology, Universiti Kebangsaan Malaysia, UKM 43500 Bangi Selangor, Malaysia

## Abstract

The title mol­ecule, C_13_H_17_ClN_2_O_3_S, shows an *anti* and *syn* disposition of the butanoyl and 2,5-dimethoxyphenyl groups with respect to the thione and is stabilized by intra­molecular N—H⋯O and weak C—H⋯S hydrogen bonds. In the crystal, inter­molecular N—H⋯S hydrogen bonds link the mol­ecules into centrosymmetric dimers. The crystal structure is stabilized by weak C—H⋯O and C—H⋯S contacts.

## Related literature

For the structures of related thio­ureas, see: Yamin *et al.* (2011 ▶); Yusof *et al.* (2011 ▶).
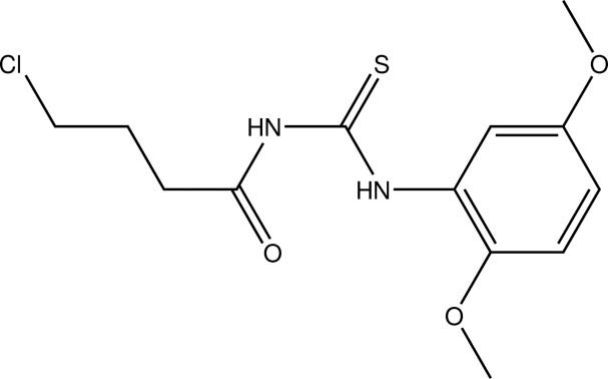


## Experimental

### 

#### Crystal data

C_13_H_17_ClN_2_O_3_S
*M* *_r_* = 316.80
Triclinic, 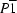

*a* = 7.6882 (18) Å
*b* = 9.151 (2) Å
*c* = 10.939 (3) Å
α = 98.536 (5)°
β = 97.787 (5)°
γ = 101.489 (5)°
*V* = 734.9 (3) Å^3^
*Z* = 2
Mo *K*α radiation
μ = 0.41 mm^−1^
*T* = 298 K
0.29 × 0.25 × 0.19 mm

#### Data collection

Bruker SMART APEX CCD area-detector diffractometer
Absorption correction: multi-scan (*SADABS*; Bruker, 2000 ▶) *T*_min_ = 0.890, *T*_max_ = 0.926
9303 measured reflections
3351 independent reflections
2928 reflections with *I* > 2σ(*I*)
*R*_int_ = 0.018

#### Refinement

*R*[*F* ^2^ > 2σ(*F* ^2^)] = 0.039
*wR*(*F* ^2^) = 0.105
*S* = 1.06
3351 reflections
181 parameters
H-atom parameters constrained
Δρ_max_ = 0.24 e Å^−3^
Δρ_min_ = −0.22 e Å^−3^

### 

Data collection: *SMART* (Bruker, 2000 ▶); cell refinement: *SAINT* (Bruker, 2000 ▶); data reduction: *SAINT*; program(s) used to solve structure: *SHELXS97* (Sheldrick, 2008 ▶); program(s) used to refine structure: *SHELXL97* (Sheldrick, 2008 ▶); molecular graphics: *SHELXTL* (Sheldrick, 2008 ▶); software used to prepare material for publication: *SHELXTL*, *PARST* (Nardelli, 1995 ▶) and *PLATON* (Spek, 2009 ▶).

## Supplementary Material

Crystal structure: contains datablock(s) global, I. DOI: 10.1107/S1600536811036002/bh2378sup1.cif

Structure factors: contains datablock(s) I. DOI: 10.1107/S1600536811036002/bh2378Isup2.hkl

Supplementary material file. DOI: 10.1107/S1600536811036002/bh2378Isup3.cml

Additional supplementary materials:  crystallographic information; 3D view; checkCIF report

## Figures and Tables

**Table 1:** Hydrogen-bond geometry (Å, °)

*D*—H⋯*A*	*D*—H	H⋯*A*	*D*⋯*A*	*D*—H⋯*A*
N2—H2*A*⋯O1	0.86	1.93	2.663 (2)	141
C7—H7*A*⋯S1	0.93	2.51	3.1853 (18)	129
N1—H1*A*⋯S1^i^	0.86	2.58	3.4058 (16)	161
C3—H3*A*⋯S1^i^	0.97	2.83	3.5633 (19)	133
C12—H12*A*⋯O2^ii^	0.96	2.50	3.259 (3)	136

Symmetry codes: (i) 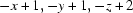; (ii) 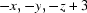.

**Fig. 1.**
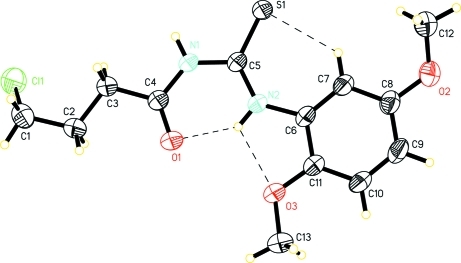
The molecular structure of the title compound, with displacement ellipsoids drawn at the 50% probability level.

**Fig. 2.**
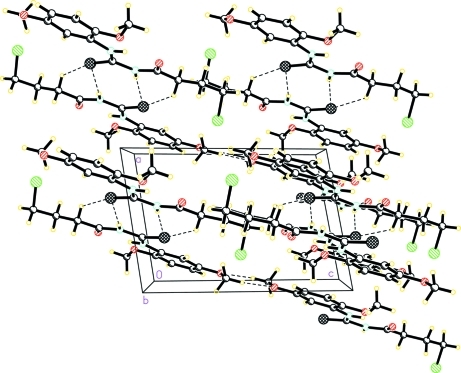
A packing diagram of the title compound viewed down the b axis. Hydrogen bonds are shown as dashed lines.

## supplementary crystallographic information

### Comment

The title compound (Fig. 1) is analogous to the previously reported *N*-(4-chlorobutanoyl)-*N*'-(2-fluorophenyl)thiourea (Yusof *et al.*, 2011) except that the methoxy groups are attached at the 2 and 5 positions of the phenyl ring. The carbonylthiourea fragment C4/O1/N1/C5/S1/N2 and the benzene ring, C6···C11, are each planar with the maximum deviation from the least-squares planes of 0.024 (2) Å for atom C4. The benzene ring and carbonylthiourea moiety form a dihedral angle of 5.67 (6)°, much smaller than angles observed in the previously reported thioureas *N*-(4-chlorobutanoyl)-*N*'-(2-fluorophenyl)thiourea [74.78 (19)° and 82.3 (2)° for two independent molecules] and *N*-(4-chlorobutanoyl)-*N*'-phenylthiourea [72.98 (12)° and 81.47 (14)° for two independent molecules] (Yusof *et al.*, 2011; Yamin *et al.*, 2011). The bond lengths and angles in the title thiourea are in normal ranges and comparable to those in the analogous compounds. The molecule maintains the *trans*-*cis* configuration with respect to the position of the butanoyl and 2,5-dimethoxyphenyl groups against the thiono C=S group bond across their C—N bonds.

The molecule is stabilized by three intramolecular contacts, N—H···O and C—H···S. In the crystal packing, the molecules are linked by N—H···S, C—H···S and C—H···O intermolecular hydrogen bonds (symmetry codes as in Table 1) and form dimers (Fig. 2).

### Experimental

A solution of 4-chlorobutanoylisothiocyanate (1.25 g, 6.33 mmol) in 30 ml of acetone was added into a flask containing 30 ml acetone solution of 2,5-dimethoxyaniline (0.82 g, 6.33 mmol). The mixture was refluxed for 1 h. Then, the solution was filtered-off and left to evaporate at room temperature. The colourless solid was obtained after one day of evaporation (yield 74%).

### Refinement

H atoms bonded to C atoms were positioned geometrically with C—H = 0.93–0.97 Å and constrained to ride on their parent atoms with *U*_iso_(H)= *xU*_eq_(parent atom) where *x*=1.5 for CH_3_ group and 1.2 for CH and CH_2_ groups. Amine H atoms were also placed in idealized positions and refined with N—H bond lengths restrained to 0.86 Å and *U*_iso_(H)= 1.2*U*_eq_(parent N atom).

### Crystal data

**Table:**

C_13_H_17_ClN_2_O_3_S	*Z* = 2
*M**_r_* = 316.80	*F*(000) = 332
Triclinic, *P*1	*D*_x_ = 1.432 Mg m^−^^3^
Hall symbol: -P 1	Mo *K*α radiation, λ = 0.71073 Å
*a* = 7.6882 (18) Å	Cell parameters from 934 reflections
*b* = 9.151 (2) Å	θ = 1.9–27.5°
*c* = 10.939 (3) Å	µ = 0.41 mm^−^^1^
α = 98.536 (5)°	*T* = 298 K
β = 97.787 (5)°	Slab, colourless
γ = 101.489 (5)°	0.29 × 0.25 × 0.19 mm
*V* = 734.9 (3) Å^3^	

### Data collection

**Table:**

Bruker SMART APEX CCD area-detector diffractometer	3351 independent reflections
Radiation source: fine-focus sealed tube	2928 reflections with *I* > 2/s(*I*)
graphite	*R*_int_ = 0.018
Detector resolution: 83.66 pixels mm^-1^	θ_max_ = 27.5°, θ_min_ = 1.9°
ω scan	*h* = −9→9
Absorption correction: multi-scan (*SADABS*; Bruker, 2000)	*k* = −11→11
*T*_min_ = 0.890, *T*_max_ = 0.926	*l* = −14→14
9303 measured reflections	

### Refinement

**Table:**

Refinement on *F*^2^	Primary atom site location: structure-invariant direct methods
Least-squares matrix: full	Secondary atom site location: difference Fourier map
*R*[*F*^2^ > 2σ(*F*^2^)] = 0.039	Hydrogen site location: inferred from neighbouring sites
*wR*(*F*^2^) = 0.105	H-atom parameters constrained
*S* = 1.06	*w* = 1/[σ^2^(*F*_o_^2^) + (0.053*P*)^2^ + 0.1878*P*] where *P* = (*F*_o_^2^ + 2*F*_c_^2^)/3
3351 reflections	(Δ/σ)_max_ < 0.001
181 parameters	Δρ_max_ = 0.24 e Å^−^^3^
0 restraints	Δρ_min_ = −0.22 e Å^−^^3^
0 constraints	

### Fractional atomic coordinates and isotropic or equivalent isotropic displacement parameters (Å^2^)

**Table:**

	*x*	*y*	*z*	*U*_iso_*/*U*_eq_	
Cl1	0.76739 (8)	0.42437 (6)	0.51080 (5)	0.06766 (17)	
S1	0.35046 (6)	0.36496 (4)	1.10956 (4)	0.04799 (14)	
O1	0.38199 (19)	0.05467 (12)	0.74925 (12)	0.0523 (3)	
O2	0.0551 (2)	−0.00809 (15)	1.36092 (13)	0.0633 (4)	
O3	0.23484 (18)	−0.21076 (12)	0.90512 (12)	0.0521 (3)	
N1	0.40933 (18)	0.27749 (13)	0.88324 (12)	0.0393 (3)	
H1A	0.4464	0.3739	0.8901	0.047*	
N2	0.29315 (17)	0.07994 (13)	0.97717 (12)	0.0379 (3)	
H2A	0.3046	0.0287	0.9073	0.045*	
C1	0.5613 (3)	0.2829 (2)	0.45811 (17)	0.0549 (4)	
H1B	0.4647	0.3319	0.4332	0.066*	
H1C	0.5743	0.2142	0.3851	0.066*	
C2	0.5121 (3)	0.19342 (19)	0.55845 (15)	0.0466 (4)	
H2B	0.6092	0.1445	0.5826	0.056*	
H2C	0.4052	0.1144	0.5235	0.056*	
C3	0.4770 (3)	0.28733 (18)	0.67463 (15)	0.0457 (4)	
H3A	0.5857	0.3630	0.7127	0.055*	
H3B	0.3837	0.3401	0.6506	0.055*	
C4	0.4193 (2)	0.19249 (17)	0.76973 (15)	0.0388 (3)	
C5	0.3481 (2)	0.22955 (16)	0.98775 (14)	0.0353 (3)	
C6	0.2192 (2)	−0.00946 (16)	1.06058 (14)	0.0356 (3)	
C7	0.1739 (2)	0.04605 (17)	1.17392 (15)	0.0414 (3)	
H7A	0.1919	0.1500	1.2009	0.050*	
C8	0.1013 (2)	−0.05441 (19)	1.24712 (15)	0.0435 (4)	
C9	0.0757 (2)	−0.20879 (19)	1.20822 (17)	0.0475 (4)	
H9A	0.0292	−0.2752	1.2584	0.057*	
C10	0.1191 (2)	−0.26447 (18)	1.09489 (17)	0.0463 (4)	
H10A	0.1010	−0.3686	1.0688	0.056*	
C11	0.1893 (2)	−0.16674 (17)	1.01961 (15)	0.0394 (3)	
C12	0.0271 (3)	0.1408 (2)	1.38725 (18)	0.0584 (5)	
H12A	−0.0043	0.1587	1.4693	0.088*	
H12B	−0.0689	0.1522	1.3260	0.088*	
H12C	0.1354	0.2125	1.3843	0.088*	
C13	0.1689 (3)	−0.36576 (19)	0.84650 (18)	0.0540 (4)	
H13A	0.2096	−0.3825	0.7675	0.081*	
H13B	0.0396	−0.3895	0.8329	0.081*	
H13C	0.2130	−0.4296	0.8997	0.081*	

### Atomic displacement parameters (Å^2^)

**Table:**

	*U*^11^	*U*^22^	*U*^33^	*U*^12^	*U*^13^	*U*^23^
Cl1	0.0734 (3)	0.0600 (3)	0.0700 (3)	0.0030 (2)	0.0293 (3)	0.0139 (2)
S1	0.0672 (3)	0.0281 (2)	0.0468 (2)	−0.00080 (17)	0.0255 (2)	0.00220 (16)
O1	0.0760 (8)	0.0283 (6)	0.0488 (7)	−0.0001 (5)	0.0206 (6)	0.0022 (5)
O2	0.1019 (11)	0.0522 (7)	0.0481 (7)	0.0223 (7)	0.0333 (7)	0.0227 (6)
O3	0.0739 (8)	0.0282 (5)	0.0537 (7)	0.0013 (5)	0.0262 (6)	0.0057 (5)
N1	0.0517 (8)	0.0240 (6)	0.0410 (7)	0.0006 (5)	0.0160 (6)	0.0058 (5)
N2	0.0488 (7)	0.0259 (6)	0.0370 (6)	0.0008 (5)	0.0123 (5)	0.0050 (5)
C1	0.0685 (12)	0.0546 (11)	0.0401 (9)	0.0094 (9)	0.0141 (8)	0.0050 (8)
C2	0.0597 (10)	0.0363 (8)	0.0413 (9)	0.0074 (7)	0.0120 (7)	0.0007 (6)
C3	0.0657 (11)	0.0325 (8)	0.0403 (8)	0.0090 (7)	0.0179 (7)	0.0059 (6)
C4	0.0438 (8)	0.0312 (7)	0.0396 (8)	0.0029 (6)	0.0103 (6)	0.0055 (6)
C5	0.0372 (7)	0.0290 (7)	0.0388 (7)	0.0027 (6)	0.0092 (6)	0.0076 (5)
C6	0.0370 (7)	0.0299 (7)	0.0395 (8)	0.0027 (6)	0.0060 (6)	0.0117 (6)
C7	0.0515 (9)	0.0320 (7)	0.0409 (8)	0.0056 (6)	0.0094 (7)	0.0113 (6)
C8	0.0517 (9)	0.0422 (8)	0.0388 (8)	0.0086 (7)	0.0097 (7)	0.0152 (7)
C9	0.0557 (10)	0.0405 (8)	0.0491 (9)	0.0047 (7)	0.0120 (8)	0.0226 (7)
C10	0.0565 (10)	0.0293 (7)	0.0527 (9)	0.0031 (7)	0.0114 (8)	0.0134 (7)
C11	0.0426 (8)	0.0313 (7)	0.0437 (8)	0.0035 (6)	0.0089 (6)	0.0095 (6)
C12	0.0805 (13)	0.0503 (10)	0.0482 (10)	0.0129 (9)	0.0235 (9)	0.0116 (8)
C13	0.0685 (12)	0.0324 (8)	0.0579 (11)	0.0048 (8)	0.0158 (9)	0.0026 (7)

### Geometric parameters (Å, °)

**Table:**

Cl1—C1	1.798 (2)	C3—C4	1.507 (2)
S1—C5	1.6750 (16)	C3—H3A	0.9700
O1—C4	1.2156 (19)	C3—H3B	0.9700
O2—C8	1.370 (2)	C6—C7	1.385 (2)
O2—C12	1.415 (2)	C6—C11	1.405 (2)
O3—C11	1.370 (2)	C7—C8	1.390 (2)
O3—C13	1.4267 (19)	C7—H7A	0.9300
N1—C4	1.3839 (19)	C8—C9	1.380 (2)
N1—C5	1.3905 (19)	C9—C10	1.378 (3)
N1—H1A	0.8600	C9—H9A	0.9300
N2—C5	1.3324 (18)	C10—C11	1.384 (2)
N2—C6	1.4149 (18)	C10—H10A	0.9300
N2—H2A	0.8600	C12—H12A	0.9600
C1—C2	1.508 (2)	C12—H12B	0.9600
C1—H1B	0.9700	C12—H12C	0.9600
C1—H1C	0.9700	C13—H13A	0.9600
C2—C3	1.513 (2)	C13—H13B	0.9600
C2—H2B	0.9700	C13—H13C	0.9600
C2—H2C	0.9700		
			
C8—O2—C12	117.89 (13)	N1—C5—S1	116.73 (10)
C11—O3—C13	117.32 (13)	C7—C6—C11	119.81 (13)
C4—N1—C5	129.36 (12)	C7—C6—N2	125.40 (13)
C4—N1—H1A	115.3	C11—C6—N2	114.78 (13)
C5—N1—H1A	115.3	C6—C7—C8	119.69 (14)
C5—N2—C6	131.35 (13)	C6—C7—H7A	120.2
C5—N2—H2A	114.3	C8—C7—H7A	120.2
C6—N2—H2A	114.3	O2—C8—C9	116.42 (14)
C2—C1—Cl1	112.05 (13)	O2—C8—C7	123.06 (15)
C2—C1—H1B	109.2	C9—C8—C7	120.50 (15)
Cl1—C1—H1B	109.2	C10—C9—C8	119.96 (15)
C2—C1—H1C	109.2	C10—C9—H9A	120.0
Cl1—C1—H1C	109.2	C8—C9—H9A	120.0
H1B—C1—H1C	107.9	C9—C10—C11	120.59 (15)
C1—C2—C3	114.17 (14)	C9—C10—H10A	119.7
C1—C2—H2B	108.7	C11—C10—H10A	119.7
C3—C2—H2B	108.7	O3—C11—C10	125.02 (14)
C1—C2—H2C	108.7	O3—C11—C6	115.55 (13)
C3—C2—H2C	108.7	C10—C11—C6	119.43 (15)
H2B—C2—H2C	107.6	O2—C12—H12A	109.5
C4—C3—C2	112.46 (13)	O2—C12—H12B	109.5
C4—C3—H3A	109.1	H12A—C12—H12B	109.5
C2—C3—H3A	109.1	O2—C12—H12C	109.5
C4—C3—H3B	109.1	H12A—C12—H12C	109.5
C2—C3—H3B	109.1	H12B—C12—H12C	109.5
H3A—C3—H3B	107.8	O3—C13—H13A	109.5
O1—C4—N1	122.66 (14)	O3—C13—H13B	109.5
O1—C4—C3	123.84 (14)	H13A—C13—H13B	109.5
N1—C4—C3	113.49 (13)	O3—C13—H13C	109.5
N2—C5—N1	115.06 (13)	H13A—C13—H13C	109.5
N2—C5—S1	128.21 (12)	H13B—C13—H13C	109.5

### Hydrogen-bond geometry (Å, °)

**Table:**

*D*—H···*A*	*D*—H	H···*A*	*D*···*A*	*D*—H···*A*
N2—H2A···O1	0.86	1.93	2.663 (2)	141
N2—H2A···O3	0.86	2.15	2.5895 (18)	112
C7—H7A···S1	0.93	2.51	3.1853 (18)	129
N1—H1A···S1^i^	0.86	2.58	3.4058 (16)	161
C3—H3A···S1^i^	0.97	2.83	3.5633 (19)	133
C12—H12A···O2^ii^	0.96	2.50	3.259 (3)	136

Symmetry codes:  (i) −*x*+1, −*y*+1, −*z*+2; (ii) −*x*, −*y*, −*z*+3.
